# Documentation of Stigmatizing Language in Electronic Health Records and Birth Outcomes

**DOI:** 10.1177/24731242251381595

**Published:** 2025-09-25

**Authors:** Jihye Kim Scroggins, Ismael Ibrahim Hulchafo, Sarah Harkins, Hans Moen, Michele Tadiello, Kenrick Cato, Anahita Davoudi, Dena Goffman, Janice James Aubey, Coretta Green, Maxim Topaz, Veronica Barcelona

**Affiliations:** ^1^ School of Nursing, University of North Carolina at Chapel Hill, Chapel Hill, North Carolina, USA.; ^2^ Columbia University School of Nursing, New York, New York, USA.; ^3^ Department of Computer Science, Aalto University, Espoo, Finland.; ^4^ Center for Community-Engaged Health Informatics and Data Science, Columbia University Irving Medical Center, New York, New York, USA.; ^5^ University of Pennsylvania School of Nursing, Philadelphia, Pennsylvania, USA.; ^6^ VNS Health, New York, New York, USA.; ^7^ Department of Obstetrics and Gynecology, Columbia University, New York, New York, USA.; ^8^ NewYork-Presbyterian Hospitals, New York, New York, USA.

**Keywords:** maternal morbidity, health disparities, natural language processing, electronic health records, health communication, bias

## Abstract

**Introduction:**

Stigmatizing language represents biases. Understanding its impact is crucial to improve perinatal health. We aimed to examine the association between stigmatizing language in electronic health records (EHR) and birth outcomes.

**Methods:**

We analyzed EHR data of patients admitted for childbirth (*n* = 18,897) between 2017 and 2019 using natural language processing at two hospitals in the United States. Independent variables were any stigmatizing language, and by category: marginalized language/identities, difficult patient, and unilateral/authoritarian decisions. Outcome variables included low-risk cesarean birth (Society for Maternal and Fetal Medicine [SMFM] and nulliparous, term, singleton, vertex [NTSV] definitions), postpartum hemorrhage, and chorioamnionitis.

**Results:**

Compared with patients with no stigmatizing language, patients with any stigmatizing language had higher odds of SMFM low-risk cesarean birth (adjusted odds ratio [aOR] = 1.36, 95% confidence interval [CI] = 1.23–1.50, *p* < 0.01), postpartum hemorrhage (aOR = 1.68, 95% CI = 1.51–1.88, *p* < 0.01), and chorioamnionitis (aOR = 1.23, 95% CI = 1.07–1.42, *p* < 0.01). Labeling patients as difficult was associated with higher odds of low-risk cesarean birth (SMFM aOR = 1.19, 95% CI = 1.07–1.33, *p* < 0.01), postpartum hemorrhage (aOR = 2.07, 95% CI = 1.85–2.30, *p* < 0.01), and chorioamnionitis (aOR = 1.33, 95% CI = 1.14–1.55, *p* < 0.01). Patients who had language from unilateral/authoritarian category had higher odds of low-risk cesarean birth (SMFM aOR = 1.46, 95% CI = 1.31–1.62, *p* < 0.01) and postpartum hemorrhage (aOR = 1.31, 95% CI = 1.17–1.46, *p* < 0.01).

**Discussion and Conclusion:**

Stigmatizing language in clinical notes was associated with birth outcomes. These findings highlight the need to improve perinatal health through examining individual behaviors and structural-level policies that reinforce bias.

## Introduction

Pregnancy-related morbidity is defined as any health condition attributed to or complicating pregnancy and childbirth that adversely affects a birthing person’s well-being or functioning.^1^ In 2022, the United States reported a pregnancy-related mortality ratio of 22.3 deaths per 100,000 live births, the highest among high-income countries.^2^ Pregnancy-related morbidity also affected up to 60,000 birthing people annually in the United States.^3^ Marginalized patients have significantly higher risks of pregnancy-related morbidity and mortality, and approximately 80% of mortality and 50% of morbidity are preventable.^4,5^ Thus, addressing potential contributors to pregnancy-related morbidities such as cesarean birth, postpartum hemorrhage, and chorioamnionitis may be important to improve birth outcomes in the United States.

Cesarean birth is associated with higher risks of complications including infection, excessive bleeding, and future pregnancy issues such as placenta accreta, placenta previa, and uterine rupture.^6–8^ Cesarean birth is also associated with longer hospital stays and higher health care costs.^9^ Therefore, it is crucial to reduce unnecessary cesarean births, especially in low-risk pregnancies, to minimize morbidity and improve patient outcomes. Cesarean birth rates among low-risk patients range from approximately 12% to 26%, depending on how “low-risk” is defined across studies.^10^ Despite the reported variability in the prevalence of low-risk cesarean births, there are significant and persistent disparities. Racially and ethnically minoritized birthing people have significantly higher odds of low-risk cesarean birth than White women.^11,12^

Postpartum hemorrhage is the leading cause of severe pregnancy-related morbidity. Research on national data indicates that approximately 3% of birth hospitalizations from 2000 to 2019 were complicated by a postpartum hemorrhage.^13^ A more recent study examined quantitative blood loss (QBL) at a tertiary level hospital and reported that hemorrhage occurred in up to 25% of postpartum patients.^14^ This discrepancy has been attributed to differences in patient populations and inclusion criteria, such as the use of QBL,^14^ which has been shown to be more accurate than visual estimation of blood loss.^15^ In addition, Black patients have up to 2.5 times higher odds of postpartum hemorrhage than White patients.^16^

Chorioamnionitis, an infection of the amniotic fluid and membranes surrounding the fetus during pregnancy, also significantly contributes to pregnancy-related morbidity.^14^ This condition can lead to complications such as postpartum hemorrhage due to uterine atony and sepsis.^17^ Chorioamnionitis affects up to 6% of pregnancies in the United States.^18^ Furthermore, chorioamnionitis is more prevalent among Black patients than White patients and is associated with higher rates of cesarean birth.^19^

Racism and bias are among the primary drivers of pregnancy-related inequities. Qualitative studies have described the negative experiences of minoritized patients in pregnancy and birth settings. These patient experiences include feeling unheard, a lack of autonomy, and poor communication.^20–22^ Clinician bias has been documented in research as well, resulting in differential treatment of cardiovascular diseases, pain, and surgical outcomes.^23–25^ Clinician documentation in electronic health records (EHR) has been shown to reflect unconscious biases and stereotypes, which may negatively affect the quality of care provided.^26^ One type of clinician bias in written documentation that has received increased attention in recent research is that of stigmatizing language.

Stigmatizing language can convey unintended meanings and reinforce socially constructed power dynamics, which disproportionately affect patients with marginalized identities.^27^ Several categories of stigmatizing language in clinician documentation have been identified, including portraying patients as difficult, using quotations to indicate disbelief, expressing disapproval of patient actions, and centering clinician authority.^28,29^ The presence of stigmatizing language in the EHR can perpetuate biases and negative attitudes among health care providers.^30^ One study found that resident physicians and medical students who read notes containing stigmatizing language employed less aggressive pain management for patients.^26^ Stigmatizing language has also been associated with higher diagnostic error rates among patients who died while hospitalized.^31^ Disparities have also been noted in stigmatizing language use by race and ethnicity among patients hospitalized for labor and birth.^32^ Additionally, clinicians who used nonstigmatizing, guideline-consistent language in patient notes were more likely to have documented plans for continued care.^33^

The relationship between stigmatizing language in clinical documentation and pregnancy-related morbidities remains poorly understood. This gap is significant, as patients who are Black and insured by Medicaid often experience adverse maternal health outcomes, partly due to explicit and implicit biases in health care.^34^ Analyzing the language used in clinical notes during the hospital birth admission could shed light on how such language could be a marker reflecting quality of care, leading to poorer outcomes. This could potentially highlight important opportunities to interrupt biases and improve the quality of perinatal care. Therefore, the purpose of this study was to examine the association between the use of stigmatizing language in clinical documentation and the occurrence of low-risk cesarean birth, postpartum hemorrhage, and chorioamnionitis.

### Theoretical framework

**FIG. 1.**
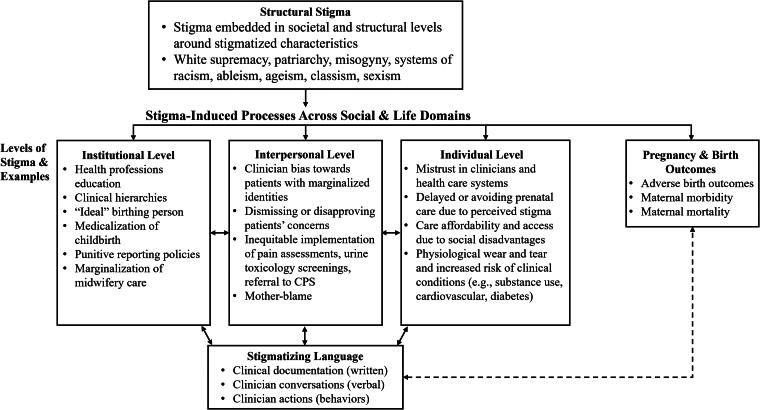
Adapted conceptual model of the study. We adapted the theory of structural stigma^35,36^ and the structural racism and inequities in maternal health framework^37^ to demonstrate the multiplate pathways through which stigma influences pregnancy and birth outcomes. Structural stigma is the primary cause of perinatal health inequities. Stigmatizing language is a marker of bias that manifests across institutional, interpersonal, and individual levels. The dashed line represents the hypothesized associations between stigmatizing language and birth outcomes.

We present the conceptual model of the current study in Figure 1, which was adapted from the theory of structural stigma^35,36^ and the structural racism and inequities in maternal health framework,^37^ to demonstrate the multiple pathways through which stigma influences pregnancy and birth outcomes. The theory of structural stigma posits that structural stigma embedded in societal and structural levels influences one’s financial, environmental, and social opportunities and resources across the life course.^36^ Structural stigma is associated with adverse health outcomes through “stigma-induced processes,” which perpetuate stigma across social and life domains, including health care systems^36^ at institutional, interpersonal, and individual levels. The structural racism and inequities in maternal health conceptual framework identifies bias as a manifestation of structural racism, which results in increased exposure to psychosocial stressors and contributes to disproportionately high rates of maternal morbidity and mortality among racially and ethnically minoritized patients.^37^

In the adapted conceptual model (Fig. 1), we identify structural stigma, such as the legacies of White supremacy, patriarchy, misogyny, and systemic racism, as the fundamental drivers of perinatal health inequities in the United States.^38^ These structural systems shape societal norms that stigmatize patients with marginalized identities that fall outside of what the dominant culture perceives to be “preferred” in perinatal care.^39^ These identities can be based on various characteristics, including employment, skin color, primary language, education, marital status, body size, and disability across institutional, interpersonal, and individual levels.^39^

At the institutional level, we highlight examples demonstrating how stigma and bias operate in the field of obstetric medicine, including the marginalization of midwives as primary birth attendants,^40^ the rise of a medicalized childbirth model dominated by White physicians,^41^ and harmful assumptions of a “default” birthing person.^42^ At the interpersonal level, clinician bias toward patients with marginalized identities can present as the dismissal of patients’ pain or pregnancy concerns,^43^ unequal implementation of protocols for postpartum pain assessments and toxicology screenings,^44,45^ disproportionate referrals to child protective services,^46^ and mother-blaming assumptions, in which clinicians perceive negative infant outcomes as a consequence of maternal behaviors.^47^ Stigma at the structural, institutional, and interpersonal levels influences the individual level, where stigma is internalized and manifested in ways, such as mistrust in clinicians and health care systems,^48^ delayed or avoided prenatal care,^49^ limited affordability and access to services,^50,51^ epigenetic changes,^52^ and increased risk of comorbid conditions.^53^ Therefore, stigma is compounded across multiple levels in society and increases the risk of adverse pregnancy and birth outcomes.^39,54^

Stigmatizing language is a marker of bias that is communicated through written documentation, verbal language, and clinician behaviors in health care systems.^39^ In the adapted model, we conceptualize that stigma at structural, institutional, and interpersonal levels is transmitted through the documentation of stigmatizing language in clinical notes. For example, health professions education reflects institutional bias by promoting documentation practices that emphasize unnecessary social risk factors and quote patients in ways that can convey judgments or disbelief.^29^ Stigmatizing language can also represent interpersonal bias, such as clinician attitudes and assumptions toward patients.^28^ In addition, patients with marginalized identities based on social, behavioral, and clinical characteristics are more likely to have stigmatizing language documented, including by race and ethnicity, socioeconomic status, and complexity or severity of medical conditions.^32,55^ Therefore, in applying this model to our study, we hypothesize that stigmatizing language is one pathway through which stigma and bias contribute to adverse birth outcomes.

## Methods

### Data and study settings

**FIG. 2.**
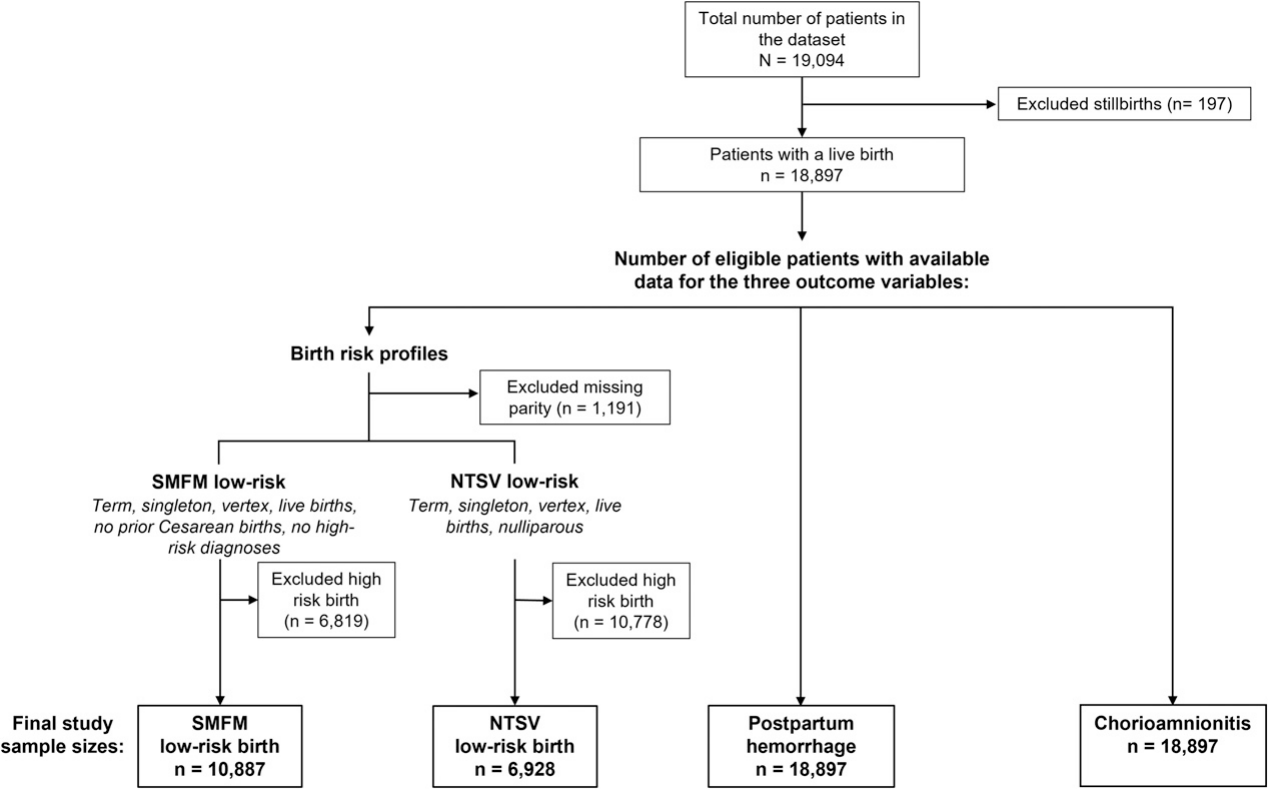
Sample size flowchart. High-risk diagnoses include: preexisting diabetes mellitus; gestational diabetes mellitus; abnormal glucose tolerance; placental accreta; placenta increta; placenta percreta; placental infarction; placenta previa; placental abruption; retained placenta; placental polyp; placental insufficiency; malformation of placenta; other placental conditions; human immunodeficiency viruses; eclampsia; hemolysis, elevated liver enzymes, and low platelets (HELLP) syndrome; circulatory conditions (e.g., cerebral venous thrombosis in pregnancy), attempted application of vacuum extractor and forceps. For the full list of high-risk conditions and corresponding ICD codes, see Armstrong et al.^56^ NTSV, nulliparous, term, singleton, vertex; SMFM, Society of Maternal-Fetal Medicine.

We conducted a cross-sectional study to examine stigmatizing language and birth outcomes in the notes of patients greater than 20 weeks’ gestation who were admitted for childbirth between 2017 and 2019 at two large metropolitan hospitals in the Northeastern United States. A total of 19,094 patients met the initial study inclusion criteria and had at least one free-text clinical note in their EHR (Fig. 2). Outcome variables (low-risk cesarean birth, postpartum hemorrhage, and chorioamnionitis) were created using the 10th revision of the International Classification of Diseases (ICD-10) diagnosis and procedural codes, and data pulled from EHR and billing systems and clinical note queries. Patients who were missing parity and those with high-risk pregnancies^56^ were excluded from the cesarean birth outcome according to the respective low-risk definitions. The final sample size for each clinical outcome differed based on these restrictions and definitions. This research adhered to ethical guidelines and received approval from the Institutional Review Board at Columbia University Medical Center (AAAT9870).

### Primary exposure and outcome variables

We used our previously developed and well-performing natural language processing (NLP) text classification model^57^ to analyze clinical notes for the current study. The primary study exposure was the presence of stigmatizing language in patient notes. This was defined as any stigmatizing language, as well as four specific stigmatizing language categories informed by prior qualitative research (marginalized language/identities, difficult patient, and unilateral/authoritarian decisions).^29^ The marginalized language/identities category captures superfluous documentation of societal and behavioral factors reflecting the marginalization of people with specific demographic characteristics. This category is characterized by unfavorable language that may reinforce negative stereotypes (e.g., “unregistered,” “substance abuse,” “unemployed”). Difficult patient language refers to characterizations of patients as noncompliant or problematic such as “poor effort with pushing.” The unilateral/authoritarian decisions category emphasizes the dominance of clinicians’ decisions, often overshadowing patient autonomy. More examples of these languages in clinical notes can be found in the Supplementary Appendix.

We focused on three primary outcome variables: low-risk cesarean birth, postpartum hemorrhage, and chorioamnionitis. Although there is no universal gold standard definition of “low-risk” cesarean birth, one commonly used criterion is nulliparous, term pregnancy, singleton gestation, vertex (NTSV) presentation.^58^ This definition does not account for maternal or fetal health conditions that can impact the likelihood of an indicated cesarean birth. Alternatively, the Society for Maternal-Fetal Medicine (SMFM) defines low-risk cesarean births as those involving a singleton, term, vertex pregnancy, regardless of parity, and excludes cases with some high-risk diagnoses such as placenta previa, eclampsia, and a history of prior cesarean.^56^ Thus, we examined both SMFM and NTSV definitions of low-risk cesarean birth. We defined postpartum hemorrhage by the presence of any of the following criteria: ICD-10 diagnostic codes, any blood transfusion, peripartum hysterectomy, use of an intrauterine balloon tamponade device, or estimated blood loss exceeding 1000 mL.^59^ Chorioamnionitis was identified through relevant ICD-10 diagnostic codes.

We adjusted for confounding by including several covariates in multivariable logistic regression models: patient race and ethnicity, insurance type, age, marital status, obesity, chronic hypertension, gestational hypertension, and preeclampsia. Missing race and ethnicity were imputed using previously described procedures, based on patient surname and geocoded home address.^60^ We also adjusted for gestational age at birth, multiple gestation, and parity as covariates in models for postpartum hemorrhage and chorioamnionitis. Gestational diabetes was also included in models for NTSV, postpartum hemorrhage, and chorioamnionitis.

### Statistical analysis

Descriptive statistics (frequency and percentage) of the study sample were calculated and reported to describe sample characteristics. We performed bivariate analyses (chi-square tests) to examine unadjusted associations between stigmatizing language and birth outcomes. We employed multivariable logistic regression to examine adjusted associations for each outcome variable (low-risk cesarean birth, postpartum hemorrhage, and chorioamnionitis). For low-risk cesarean birth, we built two separate models for the SMFM and NTSV definitions of low-risk cesarean. We also built two separate models for postpartum hemorrhage. The first model used the main definition of postpartum hemorrhage as defined above, and the second included cases meeting the main definition and/or where uterotonic medications, such as carboprost tromethamine, misoprostol, or methylergonovine, were administered. The second postpartum hemorrhage model was included as these medications are used both for managing excessive bleeding and for prophylactic purposes.^61^ Previous studies have adopted similar definitions to account for the therapeutic and preventive roles of these medications.^14^ In addition, we conducted additional analyses to explore potential interaction effects between stigmatizing language and (1) race and ethnicity and (2) insurance type on birth outcomes in separate models. We used an alpha of 0.05 to indicate statistical significance. All analyses were performed using Python in JupyterLab 3.0.

## Results

The final study sample sizes were 10,887 (SMFM) and 6928 (NTSV) for low-risk cesarean birth, and 18,897 patients for postpartum hemorrhage and chorioamnionitis. The frequencies of birth outcomes in the study sample are presented in Table 1. Nearly one in five patients had cesarean births using the SMFM definition, and approximately one in three patients met criteria for the NTSV definition. Approximately 9% of patients had a postpartum hemorrhage, and less than 5% of patients had chorioamnionitis.

**Table 1.** Frequencies of Birth Outcomes and Characteristics of Patients Admitted for Labor and Birth by Birth Outcome

	Low-risk cesarean birth		Postpartum hemorrhage and chorioamnionitis
	SMFM	NTSV	Full sample who had a live birth
	*n* = 10,887	*n* = 6928	*n* = 18,897
	***n* (%)**	***n* (%)**	***n* (%)**
Frequencies of birth outcomes			
Low-risk cesarean birth, SMFM	2148 (19.73)	—	—
Low-risk cesarean birth, NTSV	—	2150 (31.03)	—
Postpartum hemorrhage	—	—	1778 (9.41)
Chorioamnionitis	—	—	913 (4.83)
Race and ethnicity			
White	2485 (22.82)	1728 (24.94)	4284 (22.67)
Black	1188 (10.91)	711 (10.26)	2138 (11.31)
Hispanic	6439 (59.14)	3,918 (56.55)	11,147 (58.99)
API	683 (6.27)	524 (7.56)	1181 (6.25)
AI/AN	4 (0.04)	3 (0.04)	10 (0.05)
Multiracial	11 (0.10)	6 (0.09)	16 (0.08)
Missing	77 (0.71)	38 (0.55)	121 (0.64)
Maternal age			
13–19	529 (4.86)	496 (7.16)	681 (3.60)
20–34	7962 (73.13)	5198 (75.03)	13,004 (68.81)
≥35	2396 (22.01)	1234 (17.81)	5212 (27.58)
Marital status			
Single	6429 (59.05)	4011 (57.89)	10,786 (57.07)
Married	4174 (38.33)	2720 (39.26)	7618 (40.31)
Divorced	30 (0.28)	13 (0.19)	89 (0.47)
Widowed	7 (0.06)	3 (0.04)	11 (0.06)
Other^a^	219 (2.01)	167 (2.41)	353 (1.87)
Missing	28 (0.26)	14 (0.20)	40 (0.21)
Insurance type			
Medicaid	6129 (56.30)	3553 (51.28)	10,674 (56.48)
Private	4758 (43.70)	3375 (48.71)	8123 (42.98)
Missing	0 (0.00)	0 (0.00)	100 (0.53)
Parity			
Nulliparous	6128 (56.29)	6928 (100.00)	8189 (43.33)
Multiparous	4759 (43.71)	0 (0.00)	9517 (50.36)
Missing	0 (0.00)	0 (0.00)	1191 (6.30)
Mode of birth			
Vaginal	8739 (80.27)	4778 (68.97)	11,657 (61.69)
Cesarean	2148 (19.73)	2150 (31.03)	7240 (38.31)
Gestational age at birth			
Term (≥37 weeks)	10,887 (100.00)	6928 (100.00)	16,802 (88.91)
Preterm (<37 weeks)	0 (0.00)	0 (0.00)	2095 (11.09)
Any stigmatizing language	5025 (46.16)	3352 (48.38)	9333 (49.39)
Marginalized language/identities	1071 (9.84)	703 (10.23)	1719 (9.10)
Difficult patient	2668 (24.51)	1847 (26.66)	5415 (28.65)
Unilateral/authoritarian decisions	2532 (23.26)	1685 (24.32)	4915 (26.01)

^a^ Unspecified or unknown status.

AIAN, American Indian and Alaskan Native; API, Asian and Pacific Islander; NTSV, nulliparous, term, singleton, vertex; SMFM, Society of Maternal-Fetal Medicine.

Sample characteristics for each outcome are presented in Table 1. Among patients who had a live birth (*n* = 18,897), half were multiparous (50.36%), almost two-thirds had a vaginal birth (61.69%), and most births occurred at term (88.91%). Approximately 59% of patients were Hispanic (any race), followed by 23% White and 11% Black. Most patients were between 20 and 34 years old (68.81%), and about 28% were 35 years or older. More than half of patients were insured by Medicaid (56.48%) and were single (57.07%).

Bivariate associations between stigmatizing language and birth outcomes are presented in Table 2. Documentation of any stigmatizing language was significantly associated with all outcomes (*p* < 0.01). For specific stigmatizing language categories, documentation of difficult patient and unilateral/authoritarian decisions was also associated with most of the birth outcomes. For example, difficult patient language was more frequently documented among patients who had cesarean births (SMFM = 28.21%, NTSV = 29.49%) compared with patients who did not have cesarean births (SMFM = 23.60%, NTSV = 25.39%). Similarly, unilateral/authoritarian decision language was more frequently documented among patients who had cesarean births (SMFM = 29.19%, NTSV = 28.56%) than those who did not (SMFM = 21.80%, NTSV = 22.42%). Documentation of difficult patient language was more commonly found among patients with chorioamnionitis (34.50%) than those who did not have chorioamnionitis (28.36%).

**Table 2.** Chi-Square Results of Associations Between Stigmatizing Language and Birth Outcomes

	Cesarean birth					
	SMFM			NTSV		
	Yes	No		Yes	No	
	*n* = 2,148	*n* = 8,739		*n* = 2150	*n* = 4778	
	***n* (%)**	***n* (%)**	*p*-value	***n* (%)**	***n* (%)**	*p*-value
Any stigmatizing language	1131 (52.65)	3894 (44.56)	<0.01	1136 (52.84)	2216 (46.38)	<0.01
Marginalized language/identities	215 (10.01)	856 (9.80)	0.80	208 (9.67)	501 (10.49)	0.32
Difficult patient	606 (28.21)	2062 (23.60)	<.01	634 (29.49)	1213 (25.39)	<0.01
Unilateral/authoritarian decisions	627 (29.19)	1905 (21.80)	<0.01	614 (28.56)	1071 (22.42)	<0.01

	**Postpartum hemorrhage**		
	**Yes**	**No**	
	*n* = 1,778	*n* = 17,119	
	***n* (%)**	***n* (%)**	*p*-value
Any stigmatizing language	1129 (63.50)	8204 (47.92)	<0.01
Marginalized language/identities	140 (7.87)	1579 (9.22)	0.07
Difficult patient	833 (46.85)	4582 (26.77)	<0.01
Unilateral/authoritarian decisions	594 (33.41)	4321 (25.24)	<0.01

	**Chorioamnionitis**		
	**Yes**	**No**	
	*n* = 913	*n* = 17,984	
	***n* (%)**	***n* (%)**	*p*-value
Any stigmatizing language	498 (54.55)	8835 (49.13)	<0.01
Marginalized language/identities	91 (9.97)	1628 (9.05)	0.38
Difficult patient	315 (34.50)	5100 (28.36)	<0.01
Unilateral/authoritarian decisions	248 (27.16)	4667 (25.95)	0.44

Only “yes” values are reported in frequency for stigmatizing language and its categories. The denominator used was the total study sample size for each outcome. The sum of the percentages is greater than 100 because language categories are not mutually exclusive. Overall frequencies include any instances of language subcategories.

NTSV, nulliparous, term, singleton, vertex; SMFM, Society of Maternal-Fetal Medicine.

In adjusted analyses (Table 3), patients who had at least one category of stigmatizing language documented in their clinical notes had significantly higher odds for all outcomes examined (adjusted odds ratios [aORs] ranging from 1.23 to 1.68), compared with patients who did not have stigmatizing language documented. For instance, the presence of language from any of the stigmatizing language categories in clinical notes was associated with a significantly higher odds of experiencing postpartum hemorrhage (aOR = 1.68, 95% confidence interval [CI] = 1.51–1.88, *p* < 0.01).

**Table 3.** Binomial Multivariable Logistic Regression Analysis Results Examining Adjusted Associations Between Stigmatizing Language and Birth Outcomes

	Cesarean birth			
	SMFM		NTSV	
	aOR (95% CI)	*p*-value	aOR (95% CI)	*p*-value
No stigmatizing language	Reference		Reference	
Any stigmatizing language	1.36 (1.23, 1.50)	<0.01	1.34 (1.20, 1.49)	<0.01
Marginalized language/identities	1.03 (0.87, 1.22)	0.71	1.10 (0.91, 1.33)	0.33
Difficult patient	1.19 (1.07, 1.33)	<0.01	1.18 (1.05, 1.32)	0.01
Unilateral/authoritarian decisions	1.46 (1.31, 1.62)	<0.01	1.38 (1.23, 1.56)	<0.01

	**Postpartum hemorrhage**	
	**aOR (95% CI)**	*p*-value
No stigmatizing language	Reference	
Any stigmatizing language	1.68 (1.51, 1.88)	<0.01
Marginalized language/identities	0.82 (0.68, 1.00)	0.05
Difficult patient	2.07 (1.85, 2.30)	<0.01
Unilateral/authoritarian decisions	1.31 (1.17, 1.46)	<0.01

	**Chorioamnionitis**			
	**aOR (95% CI)**	*p*-value		
No stigmatizing language	Reference			
Any stigmatizing language	1.23 (1.07, 1.42)	<0.01		
Marginalized language/identities	1.03 (0.81, 1.32)	0.81		
Difficult patient	1.33 (1.14, 1.55)	<0.01		
Unilateral/authoritarian decisions	1.11 (0.95, 1.30)	0.21		

Covariates included in all regression models were obesity, chronic hypertension, gestational hypertension, pre-eclampsia, race and ethnicity, maternal age, marital status, and insurance type. Preterm birth, multiple gestation, and parity were added as additional covariates for postpartum hemorrhage and chorioamnionitis. Gestational diabetes was also included for NTSV, postpartum hemorrhage, and chorioamnionitis.

aOR, adjusted odds ratio; CI, confidence interval; NTSV, nulliparous, term, singleton, vertex; SMFM, Society of Maternal Fetal Medicine.

Among the specific categories of stigmatizing language, both difficult patient and unilateral/authoritarian decisions generally showed significantly higher odds for all outcomes examined. For instance, the presence of language from the unilateral/authoritarian decision category was associated with significantly higher odds of experiencing cesarean birth (SMFM aOR = 1.46, 95% CI = 1.31–1.62, *p* < 0.01 and NTSV aOR = 1.38, 95% CI = 1.23–1.56, *p* < 0.01). Similarly, documentation of difficult patient language was also associated with significantly higher odds of postpartum hemorrhage (aOR = 2.07, 95% CI = 1.85–2.30, *p* < 0.01). Similar findings were also observed for the expanded definition of hemorrhage (Supplementary Appendix).

**FIG. 3.**
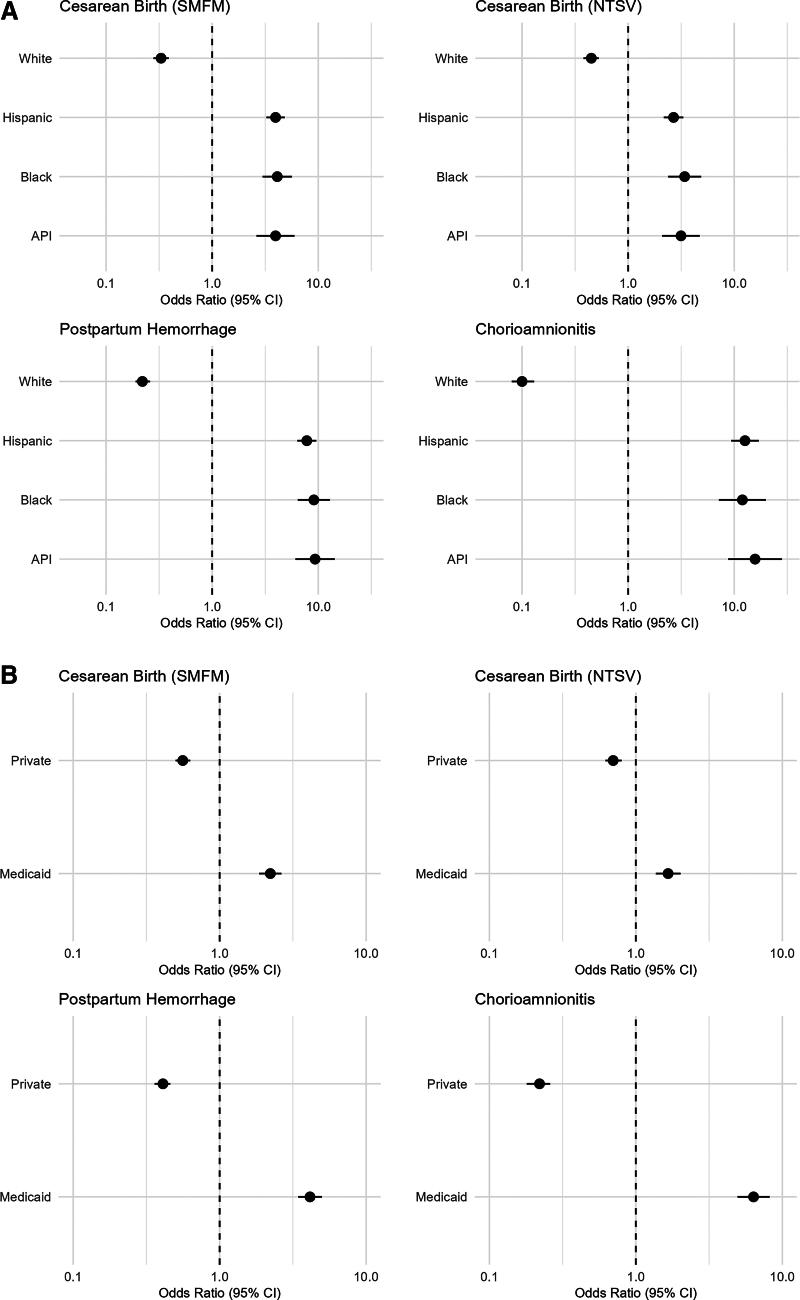
**(A)** Interaction effects between documentation of any stigmatizing language and race and ethnicity on birth outcomes. **(B)** Interaction effects between documentation of any stigmatizing language and insurance type on birth outcomes. Forest plots depicting interaction effects between documentation of any stigmatizing language and (1) race and ethnicity (Fig. 3A) and (2) insurance types (Fig. 3B) on birth outcomes. Adjusted odds ratios and 95% confidence intervals are shown on a log scale along the X-axis. CI, confidence intervals; NTSV, nulliparous, term, singleton, vertex; SMFM, Society of Maternal-Fetal Medicine.

We present the forest plots, which depict the interaction effects between documentation of any stigmatizing language and (1) race and ethnicity and (2) insurance types on birth outcomes in Figure 3, with aORs and 95% CIs. Detailed results for all categories of stigmatizing language are shown in Tables 4 and 5. Interactions between stigmatizing language and race and ethnicity were significant for most birth outcomes (Fig. 3A, Table 4). Specifically, we found that documentation of any stigmatizing language was associated with significantly higher odds of cesarean birth for Black patients (SMFM aOR = 4.11, 95% CI = 2.99–5.65, *p* < 0.01) compared with White patients with no stigmatizing language. Hispanic patients with at least one category of stigmatizing language documented in their notes also had significantly higher odds of postpartum hemorrhage (aOR = 7.80, 95% CI = 6.36–9.58; *p* < 0.01). Similarly, there were significant interaction effects between stigmatizing language and health insurance types for most birth outcomes (Fig. 3B, Table 5). For example, patients who were insured by Medicaid whose clinical notes included any stigmatizing language had significantly higher odds of chorioamnionitis (aOR = 6.36, 95% CI = 4.95–8.19, *p* < 0.01) than patients who had private insurance with no stigmatizing language.

**Table 4.** Multivariable Logistic Regression Models Examining Interactions Between Stigmatizing Language Categories and Race and Ethnicity on Birth Outcomes

	Cesarean birth				
	SMFM		NTSV		
	aOR (95% CI)	*p*-value	aOR (95% CI)	*p*-value	
Marginalized language/identities	0.34 (0.22, 0.53)	<0.01	0.48 (0.30, 0.76)	0.03	
Marginalized language/identities * API	3.34 (1.18, 9.49)	0.02	3.15 (1.13, 8.73)	<0.01	
Marginalized language/identities * Black	3.10 (1.68, 5.72)	<0.01	2.88 (1.49, 5.54)	<0.01	
Marginalized language/identities * Hispanic	3.24 (1.99, 5.28)	<0.01	2.21 (1.32, 3.69)	<0.01	
Difficult patient	0.37 (0.30, 0.45)	<0.01	0.52 (0.43, 0.65)	<0.01	
Difficult patient * API	3.36 (2.08, 5.45)	<0.01	2.59 (1.63, 4.11)	<0.01	
Difficult patient * Black	3.49 (2.42, 5.04)	<0.01	2.75 (1.84, 4.10)	<0.01	
Difficult patient * Hispanic	3.03 (2.36, 3.88)	<0.01	1.92 (1.48, 2.48)	<0.01	
Unilateral/authoritarian decisions	0.36 (0.29, 0.44)	<0.01	0.45 (0.36, 0.55)	<0.01	
Unilateral/authoritarian decisions * API	4.04 (2.52, 6.48)	<0.01	3.61 (2.26, 5.79)	<0.01	
Unilateral/authoritarian decisions * Black	4.09 (2.82, 5.92)	<0.01	3.12 (2.07, 4.71)	<0.01	
Unilateral/authoritarian decisions * Hispanic	4.05 (3.17, 5.16)	<0.01	3.06 (2.36, 3.98)	<0.01	

	**Postpartum hemorrhage**	
	**aOR (95% CI)**	*p*-value
Marginalized language/identities	0.23 (0.14, 0.37)	<0.01
Marginalized language/identities * API	2.98 (0.81, 10.90)	0.10
Marginalized language/identities * Black	3.59 (1.78, 7.26)	<0.01
Marginalized language/identities * Hispanic	4.12 (2.40, 7.07)	<0.01
Difficult patient	0.31 (0.26, 0.37)	<0.01
Difficult patient * API	6.73 (5.38, 8.41)	<0.01
Difficult patient * Black	8.96 (6.35, 12.64)	<0.01
Difficult patient * Hispanic	6.73 (5.38, 8.41)	<0.01
Unilateral/authoritarian decisions	0.26 (0.21, 0.31)	<0.01
Unilateral/authoritarian decisions * API	4.38 (2.71, 7.06)	<0.01
Unilateral/authoritarian decisions * Black	6.11 (4.27, 8.74)	<0.01
Unilateral/authoritarian decisions * Hispanic	5.21 (4.10, 6.64)	<0.01

	**Chorioamnionitis**	
	**aOR (95% CI)**	*p*-value
Marginalized language/identities	0.10 (0.04, 0.24)	<0.01
Marginalized language/identities * API	13.52 (2.90, 63.12)	<0.01
Marginalized language/identities * Black	18.85 (6.36, 55.87)	<0.01
Marginalized language/identities * Hispanic	11.93 (4.61, 30.82)	<0.01
Difficult patient	0.17 (0.13, 0.24)	<0.01
Difficult patient * API	7.10 (3.64, 13.82)	<0.01
Difficult patient * Black	6.91 (3.93, 12.15)	<0.01
Difficult patient * Hispanic	8.25 (5.81, 11.72)	<0.01
Unilateral/authoritarian decisions	0.16 (0.11, 0.21)	<0.01
Unilateral/authoritarian decisions * API	8.73 (4.49, 16.97)	<0.01
Unilateral/authoritarian decisions * Black	7.19 (4.61, 9.65)	<0.01
Unilateral/authoritarian decisions * Hispanic	6.67 (4.61, 9.65)	<0.01

The asterisk (*) indicates the interaction term.Reference group was White patients with no stigmatizing language.

Model constructions.

(1) SMFM = SL + race and ethnicity + (SL × race and ethnicity) + obesity + chronic hypertension + gestational hypertension + pre-eclampsia + maternal age + marital status + insurance type.

(2) NTSV = SL + race and ethnicity + (SL × race and ethnicity) + obesity + chronic hypertension + gestational hypertension + pre-eclampsia + maternal age + marital status + insurance type + gestational diabetes + gestational diabetes.

(3) Postpartum hemorrhage = SL + race and ethnicity + (SL × race and ethnicity) + obesity + chronic hypertension + gestational hypertension + pre-eclampsia + maternal age + marital status + insurance type + preterm birth + multiple gestation + parity + gestational diabetes.

(4) Chorioamnionitis = SL + race and ethnicity + (SL × race and ethnicity) + obesity + chronic hypertension + gestational hypertension + pre-eclampsia + maternal age + marital status + insurance type + preterm birth + multiple gestation + parity + gestational diabetes.

aOR, adjusted odds ratio; API, Asian and Pacific Islander; CI, confidence interval; NTSV, nulliparous, term, singleton, vertex; SMFM, Society of Maternal-Fetal Medicine; SL, stigmatizing language.

**Table 5.** Multivariable Logistic Regression Models Examining Interactions Between Stigmatizing Language Categories and Health Insurance Type on Birth Outcomes

	Cesarean birth			
	SMFM		NTSV	
	aOR (95% CI)	*p*-value	aOR (95% CI)	*p*-value
Marginalized language/identities	0.57 (0.41, 0.79)	<0.01	0.76 (0.54, 1.08)	0.12
Marginalized language/identities* Medicaid	1.85 (1.26, 2.70)	<0.01	1.41 (0.94, 2.11)	0.10
Difficult patient	0.62 (0.54, 0.73)	<0.01	0.77 (0.66, 0.90)	<.01
Difficult patient* Medicaid	1.73 (1.40, 2.13)	<0.01	1.29 (1.04, 1.62)	0.02
Unilateral/authoritarian decisions	0.64 (0.55, 0.74)	<0.01	0.75 (0.64, 0.88)	<.01
Unilateral/authoritarian decisions* Medicaid	2.23 (1.81, 2.74)	<0.01	1.76 (1.40, 2.21)	<.01

	**Postpartum hemorrhage**	
	**aOR (95% CI)**	*p*-value
Marginalized language/identities	0.32 (0.22, 0.48)	<0.01
Marginalized language/identities* Medicaid	2.74 (1.73, 4.34)	<0.01
Difficult patient	0.64 (0.55, 0.73)	<0.01
Difficult patient* Medicaid	3.32 (2.73, 4.03)	<0.01
Unilateral/authoritarian decisions	0.44 (0.38, 0.51)	<0.01
Unilateral/authoritarian decisions* Medicaid	2.94 (2.38, 3.63)	<0.01

	**Chorioamnionitis**			
	**aOR (95% CI)**	*p*-value		
Marginalized language/identities	0.34 (0.20, 0.58)	<0.01		
Marginalized language/identities* Medicaid	3.18 (1.77, 5.72)	<0.01		
Difficult patient	0.33 (0.26, 0.41)	<0.01		
Difficult patient* Medicaid	4.33 (3.24, 5.80)	<0.01		
Unilateral/authoritarian decisions	0.25 (0.20, 0.32)	<0.01		
Unilateral/authoritarian decisions* Medicaid	4.65 (3.40, 6.35)	<0.01		

The asterisk (*) indicates an interaction term.Reference group was patients who had private insurance with no stigmatizing language.

Model constructions.

(1) SMFM = SL + insurance type + (SL × insurance type) + obesity + chronic hypertension + gestational hypertension + pre-eclampsia + maternal age + marital status + race and ethnicity.

(2) NTSV = SL + insurance type + (SL × insurance type) + obesity + chronic hypertension + gestational hypertension + pre-eclampsia + maternal age + marital status + race and ethnicity + gestational diabetes + gestational diabetes.

(3) Postpartum hemorrhage = SL + insurance type + (SL × insurance type) + obesity + chronic hypertension + gestational hypertension + pre-eclampsia + maternal age + marital status + race and ethnicity + preterm birth + multiple gestation + parity + gestational diabetes.

(4) Chorioamnionitis = SL + insurance type + (SL × insurance type) + obesity + chronic hypertension + gestational hypertension + pre-eclampsia + maternal age + marital status + race and ethnicity + preterm birth + multiple gestation + parity + gestational diabetes.

aOR, adjusted odds ratios; API, Asian and Pacific Islander; CI, confidence intervals; NTSV, nulliparous, term, singleton, vertex; SMFM, Society of Maternal-Fetal Medicine; SL, stigmatizing language.

## Discussion

The aim of this study was to examine associations between stigmatizing language documented in birth admission clinical notes and birth outcomes, including SMFM and NTSV low-risk cesarean birth, postpartum hemorrhage, and chorioamnionitis. We found that more than half of the patients who experienced these outcomes had at least one category of stigmatizing language documented in their clinical notes. Previous studies have also found a relatively high prevalence of stigmatizing language documented in clinical notes. For example, among patients with substance use disorders, approximately 62% had at least one clinical note with stigmatizing language documented.^62^ In emergency department settings, the prevalence of any stigmatizing language in clinical notes was high across different racial groups, ranging from 53% (Asian/Pacific Islander) to 81.2% (American Indian/Native).^63^

We also found that any stigmatizing language in clinical notes was associated with a 23–60% increase in the odds of experiencing the birth outcomes under study. Stigmatizing language reflecting difficult patient and unilateral/authoritarian decisions categories was associated with significantly higher odds of these birth outcomes, including up to nearly twice the odds of postpartum hemorrhage. Our findings align with previous research, suggesting the potential negative impact of stigmatizing language on clinical behaviors and health outcomes. For example, documentation of stigmatizing language was associated with greater disease severity among patients who have diabetes.^55^ In another study of patients in inpatient medical settings, stigmatizing language was significantly associated with suboptimal care practices, such as delays in care.^31^ In contrast, use of nonstigmatizing language (e.g., documenting substance “use” as opposed to “abuse”) was associated with better patient care, including increased treatment and specialty care referrals among patients with substance use disorders.^33^ Our study offers a significant contribution to the field by highlighting the link between the documentation of stigmatizing language and birth outcomes, which have not been explored previously.

Importantly, difficult patient and unilateral/authoritarian decisions categories were consistently associated with significantly higher odds for all outcomes examined. Documentation of language labeling the patient as difficult may reflect implicit biases in how clinicians perceive patients, which could further influence clinical practice including decision-making and quality of patient interactions.^64^ Furthermore, the association between the unilateral/authoritarian decisions category and higher odds of unexpected clinical outcomes, particularly cesarean birth, underscores the importance of shared decision-making in clinical practice. Research has shown that shared decision-making may be linked to a decrease in elective surgeries.^65^ In inpatient labor and birth settings, shared decision-making or shared responsibility between patients and clinicians may strengthen patients’ confidence to decline unnecessary or unwanted clinical interventions.^66^

We also found no significant associations between language representing the marginalized identities and the clinical outcomes under study. This may have been due to the relatively low number of instances of this language category compared with other language categories. The use of language reflecting marginalized identities can contribute to bias, discrimination, and stigma in health care settings, which could further influence patient–clinician relationships and communication.^39^ It is possible that patients who were described with marginalized language/identities may receive more scrutiny or closer attention in certain clinical cases, which may contribute to preventing adverse outcomes, or have a neutral effect. For instance, patients who were documented to be “morbidly obese,” which perpetuates weight stigma, might have received closer monitoring due to perceived risks for postpartum hemorrhage. Further research is needed to better understand these complex relationships in larger patient samples.

In addition, we identified significant interaction effects between stigmatizing language and birth outcomes by patient characteristics. Specifically, Asian and Pacific Islander, Black, and Hispanic patients with stigmatizing language documented in their clinical notes had higher odds of cesarean birth, postpartum hemorrhage, and chorioamnionitis compared with White patients without stigmatizing language. Similarly, patients insured by Medicaid had higher odds of these birth outcomes compared with privately insured patients without stigmatizing language. These findings suggest that stigmatizing language may further compound existing systems of oppression and social inequities among patients’ marginalized identities, contributing to a greater risk of adverse birth outcomes. Previous research has shown that individuals are stigmatized based on multiple marginalized identities, and this intersectional stigma is associated with worse health outcomes.^67,68^ For example, Black and Hispanic patients who have substance use disorders experience stigma related to both the drug use and their race and ethnicity, which negatively influence their access to optimal treatment options, employment opportunities, and harm reduction practices, all of which contribute to worse health outcomes.^69^ Other studies also support that the multilevel discrimination is compounded among individuals with multiple marginalized identities, which contributes to perinatal health inequities.^70,71^

Interestingly, we also found that documentation of stigmatizing language was associated with lower odds for birth outcomes among patients who were White and those who were privately insured. This finding may suggest that the structurally powered and privileged identities of patients racialized as White and those who are privately insured may be less vulnerable to the harmful effects of stigma. That is, the presence of stigmatizing language in clinical notes may not translate into negative sequelae of stigma-induced processes, such as differences in treatment options, clinician mistrust, and access to care among these subgroups of patients. For example, privately insured patients with documented language related to substance use may prompt clinicians to have more vigilant care plans or treatment (e.g., early referrals, interventions, and/or follow ups), contributing to better outcomes due to their privileged social positions, which have historically afforded them greater power, credibility, or access within health care system in the United States.^72^ These findings underscore how the impact of stigma is shaped by a broader structural context and intersectionality of multiple social identities.

### Health equity implications

Our findings suggest that the documentation of stigmatizing language in the EHR is associated with birth outcomes. Given that this is the first study to examine associations between stigmatizing language and perinatal outcomes in birth settings, our study contributes to the existing literature by uncovering potential stigma-induced processes that often go unnoticed or undetected, as asserted by the theory of structural stigma.^36^ We have identified how stigma can lead to health inequities through numerous, complex mechanisms, based on the theory of structural stigma^36^ and our adapted conceptual model. There are multiple potential pathways or reasons that may explain how and why stigmatizing language leads to or is associated with birth outcomes. For example, structural and interpersonal stigma and bias toward patients based on their social or clinical characteristics, such as clinicians’ assumptions about patients related to social disadvantage and substance use, may be reflected in the use of stigmatizing language in clinical documentation, which in turn influences birth outcomes.

While the presence of stigmatizing language in clinical documentation may not necessarily result in adverse outcomes—given that such language is a marker of bias and some individuals may have other potential protective factors (e.g., powered and privileged identities)—bias itself can also contribute to adverse birth outcomes. Perceived racism and discrimination have been positively associated with a wide range of adverse perinatal outcomes, including increased risks of preterm birth, low birth weight, small gestational age, and postpartum depression, after adjusting for socioeconomic characteristics.^54,73–76^ Therefore, while it is critically important to eliminate stigmatizing language to improve quality of care, patient–provider relationships, and health outcomes, removing stigmatizing language alone will not address the underlying bias. Structural, institutional, and interpersonal stigma and bias continue to exist in health care systems in the United States, contributing to health inequities and adverse health outcomes.

As such, it is essential to consider structural stigma as the primary mechanism driving inequities in birth outcomes. Patients with marginalized identities due to age, body size, disability, immigration status, race, or substance use are more likely to experience stigma and bias when receiving pregnancy care.^39^ For example, previous research has found that patients with substance use disorders have stigmatizing language documented in their notes, including labeling them as “difficult” or “noncompliant,” and threatening to involve child protective services.^29^ These biases are rooted in the stigma at the structural level, including White supremacy, patriarchal, ableist, ageist, and misogynist systems that reinforce the assumption of a “default” birthing person^39^ and promote the belief that people who use substances are irresponsible or unfit to parent.^77–79^ Current perinatal research also upholds these biases. For example, a recent study examined racial differences in pelvic floor muscle morphology among women of reproductive age.^80^ While likely intended to address racial disparities in pelvic floor dysfunction, the study frames race as a biological risk factor, despite race being a social construct. Such an approach is harmful because it diminishes the role of structural level factors such as racism and discrimination in perinatal care as the primary driver of inequities and reinforces assumptions and biases about innate anatomical differences among racialized individuals.

In addition, clinician bias toward people with marginalized identities can negatively affect patients’ health care decisions through declining or deferring treatments or recommended care. For example, a recent study of immigrant pregnant and postpartum patients (many of whom were undocumented) reported that participants avoided or delayed care due to fear of deportation or denial of permanent residency, and potential mistreatment based on their race or immigration status.^81^ Other studies have also found that patients with undocumented immigration status were less likely to receive adequate prenatal care.^82^ Furthermore, structural stigma can perpetuate marginalized identities and social disadvantage by constraining social opportunities and resources, which can influence the access and quality of pregnancy care (e.g., recommended prenatal care), and subsequently birth outcomes.^83^ Inadequate treatment or care and limited access to high-quality care are also risk factors of clinical conditions or morbidities that further contribute to adverse health outcomes.^84–86^

While all these factors (e.g., bias, social disadvantage, inadequate pregnancy care) may be independently associated with birth outcomes and have downstream effects that further stigmatize patients with marginalized identities, structural stigma remains the fundamental force shaping perinatal care quality and driving health inequities.^37,47,87^ The theory of structural stigma emphasizes that stigma (e.g., cultural negative norms), stigmatized characteristics (e.g., substance use), and stigma-induced processes (i.e., interpersonal bias and use of stigmatizing language) can all contribute to health inequities and adverse health outcomes.^36^ However, removing one component of pathways without addressing the fundamental stigma will not resolve health inequities.^36^ Thus, broader stigma at structural and societal levels must be addressed to break the cycle,^88^ through policy reform (e.g., nonpunitive policies related to immigration or substance use, equitable health insurance coverage),^89–92^ institutional change (e.g., antiracism and antistigma training),^93–95^ and cultural shifts (e.g., transforming negative social norms toward individuals with marginalized identities).^96,97^

The current study offers novel and important insights, which contribute to the body of knowledge in health equity research by uncovering documentation of stigmatizing language as a potential measurable marker of structural stigma and bias, and one of several potential pathways through which stigma can contribute to adverse health outcomes. Importantly, this study shifts the focus from the individual’s perception of stigma or discrimination to the presence of stigma that is embedded in society, cultural norms, and institutions, including clinical training. We previously discussed that “preferred” characteristics of birthing people exist on a societal level, based on multiple marginalized identities such as legal status, skin color, educational attainment, body size, and others.^39^ The societal biases that exist based on these characteristics are reflected in the way clinicians perceive patients and their written, verbal, and behavioral expressions of language. This work signals an important advance in health equity research as it highlights an actionable area for intervention that goes beyond individual-level discrimination, stigma, and bias.^36,88^

We acknowledge that though eliminating stigmatizing language may not fully remove underlying bias, it remains an essential step in interrupting potential stigma-induced processes. This is particularly important within health care systems and educational institutions, as this language may be embedded in the initial and continuing education of clinicians, thereby contributing to inequitable perinatal health outcomes.^30^ For example, the frequent use of stigmatizing language in clinical notes highlights the need for further training and awareness to reduce bias in clinical documentation. Clinician education on the implications of stigmatizing language use and suggestions for more neutral, nonstigmatizing language is needed. Implementation of real-time alerts to detect stigmatizing language use in EHR may offer promising solutions to mitigate the use of such language, which could help prevent downstream bias among clinicians who gather information about patients by reading previous clinical notes. It is also important to consider bias at the clinical unit and systems level, which may influence individual clinician’s documentation and practices. For example, policies for placing flags in the EHR for behavioral risks or referral to social services may contribute to over-documentation of or emphasis on marginalized identities, which can further perpetuate bias in clinical care.

Addressing stigmatizing language can also foster positive patient–clinician communication and relationships by reducing medical mistrust among patients who access their own medical records and encounter documentation of stigmatizing language.^98,99^ Patients often report feeling judged, offended, or disrespected when reading stigmatizing language (e.g., labeling or disapproval) in their own clinical notes, which can negatively influence trust and relationship with clinicians.^100^ Even when such language is used to describe clinically relevant information, using nonstigmatizing, patient-centered language is important.^98^ For example, phrases such as “poor efforts with pushing” or “intolerant of vaginal exam” can convey a negative perception of a patient’s demeanor or attitude, while also being clinician-centered, authoritative, and vague in describing the issues. Instead, this clinical observation can be expressed more neutrally and accurately, such as “patient pushing intermittently and reports difficulty with sustaining pushes due to increased pelvic pressure and exhaustion after a prolonged second stage,” or “patient reports discomfort during vaginal exam due to severe pain rated 9 out of 10.” Research indicates that both patients and clinicians view the destigmatization of clinical documentation as a key component of patient-centered care that not only helps prevent or reduce bias, but also promotes shared decision-making, medical trust, patient empowerment, and autonomy, all of which may further contribute to improving birth outcomes.^98,99^

### Strengths and limitations

The strengths of this study include a large sample of notes from a diverse inpatient birthing population, use of an established and well-performing NLP model to identify stigmatizing language, and availability of clinical data to define the pregnancy-related outcomes under study. One key limitation of this study is its cross-sectional, observational study design, which limits our ability to infer causality between stigmatizing language and birth outcomes. Thus, the findings should not be interpreted as evidence of causal relationships. In this article, we describe the hypothesized mechanisms of this relationship, based on existing theories and our adapted conceptual model, through which stigmatizing language may influence birth outcomes (e.g., clinical decision-making, communication quality, patient education). These potential mechanisms or mediating pathways warrant further investigation to improve our understanding of the relationships between stigmatizing language and birth outcomes. For example, structural stigma (e.g., negative cultural norm) related to marginalized identities (e.g., socioeconomic disadvantage) can lead to interpersonal stigma (e.g., clinician’s biased perception toward patients who are unemployed), which may manifest as stigmatizing language in clinical documentation. Individual clinicians’ biases are unmeasured in the current study, and this may be a potential confounder that warrants examination in future research.

Furthermore, given the cross-sectional design of the current study, future longitudinal research is essential to improve our understanding of how stigma-induced processes influence health outcomes over the life course. The theory of structural stigma posits that stigmatized characteristics exist in multiple layers that are accumulated in one’s life course rather than a single time point during the perinatal period.^36^ For example, structural stigma can lead to life-long constraints of social opportunities and resources, which hinders individuals to reach optimal well-being through recommended health practices and health care (e.g., adequate prenatal care). Thus, longitudinal observations of stigma, stigmatizing language, and perinatal health outcomes may illuminate the scope and direction of how the cumulative exposures to stigma over time can shape and contribute to health disparities across the life course.

Another limitation is that EHR were not designed primarily for research, and some confounders were not available in our dataset. While we adjusted for important clinical and demographic covariates, other potential confounding factors (e.g., clinician characteristics, clinician bias, and documentation policies as a system-level factor) may influence documentation patterns and/or birth outcomes and were not included due to lack of availability in the EHR. For example, we relied on insurance type as a proxy for socioeconomic status because other more direct indicators, such as education and income levels were not available. Future research should consider these potential confounders and further explore the complex relationships among these factors, stigmatizing language, and birth outcomes. In addition, our study was conducted in one hospital system in the northeast United States, which may limit the generalizability of findings to other geographic locations. The SMFM and NTSV “low-risk” definitions do not fully encompass all maternal and fetal conditions, highlighting the need for further work to establish low-risk definition that is more comprehensive and truly reflective of a low-risk population. Finally, while our NLP model demonstrated strong performance in detecting stigmatizing language in previous studies, there remains a possibility that some instances of stigmatizing language may not have been captured.

## Conclusion

This study highlights the significant association between stigmatizing language and birth outcomes. Our findings contribute to the understanding of how stigmatizing language in inpatient birth settings not only perpetuates bias but also may play a critical role in achieving optimal health outcomes. Addressing the use of stigmatizing language in clinical documentation through clinician training and shared decision-making could contribute to improving birth outcomes. Further examination of hospital policies or practices, which may contribute to or reinforce clinical bias, is also warranted. Research is needed to further explore the complex relationships between specific categories of stigmatizing language and birth outcomes as well as to evaluate potential interventions to improve both documentation practices and birth outcomes.

## Abbreviations Used

AI/AN: American Indian/Alaskan Native
aOR: Adjusted odds ratio
API: Asian and Pacific Islanders
CI: Confidence interval
CPS: Child Protective Services
Fig: Figure
HELLP: Hemolysis, elevated liver enzymes, and low platelets
HER: Electronic health records
ICD: International Classification of Diseases
mL: Milliliters
NLP: Natural language processing
NTSV: Nulliparous, transverse, singleton, vertex
QBL: Quantitative blood loss
SL: Stigmatizing language
SMFM: Society of Maternal Fetal Medicine
