# Supplementary material for: Documentation of Stigmatizing Language in Electronic Health Records and Birth Outcomes
Source: Health Equity. 2025 Sep 25;9(1):580–98. doi: 10.1177/24731242251381595 (PMC12926810; doi:10.1177/24731242251381595)
Supplement: Supplementary Appendix [file 24731242251381595_suppl_appendix.docx]

**Supplemental Tables**

**Appendix 1.** Chi-square Analysis Results Examining the Association Between Stigmatizing Language and Postpartum Hemorrhage with Uterotonics

|  | **Postpartum hemorrhage including patients who received additional uterotonics** | | |
| --- | --- | --- | --- |
|  | **Yes**  n=4,004 | **No**  n=14,893 |  |
|  | **n (%)** | **n (%)** | ***P*-value** |
| **Any stigmatizing language** | 2,225 (55.57) | 7,108 (47.73) | <.01 |
| Marginalized language/identities | 346 (8.64) | 1,373 (9.22) | 0.27 |
| Difficult patient | 1,495 (37.34) | 3,920 (26.32) | <.01 |
| Unilateral/authoritarian decisions | 1,157 (28.90) | 3,758 (25.23) | <.01 |

**Appendix 2.** Binomial Multivariable Logistic Regression Analysis Results Examining Adjusted Associations Between Stigmatizing Language and Postpartum Hemorrhage with Uterotonics

|  | **Postpartum hemorrhage including patients who received additional uterotonics** | |
| --- | --- | --- |
|  | **Adjusted odds ratio**  **(95% confidence interval)** | ***P*-value** |
| No stigmatizing language | Reference |  |
| Any stigmatizing language | 1.27 (1.17,1.37) | <.01 |
| Marginalized language/identities | 0.89 (0.78,1.02) | 0.10 |
| Difficult patient | 1.49 (1.38,1.62) | <.01 |
| Unilateral/authoritarian decisions | 1.13 (1.04,1.23) | <.01 |

**Appendix 3.** Examples of Stigmatizing Language

| **Categories** | **Examples** |
| --- | --- |
| Marginalized language/identities | “she does not use drugs or smoke but drinks 6× week”  “Patient is a 32yo [year old] Dominican unmarried unemployed female”  Restating for emphasis that is already in the checklist/form data or unnecessary patient descriptor: “toxic habits,” "financially supports self," “teen mother,”  “late registrant,” “mental retardation” |
| Difficult patient | “poor effort with pushing”  “patient complaining of lightheadedness”  “FOB [father of baby] is 23 yo [year old] unemployed and not as involved as he should be.”  “Pt [patient] emotionally distraught and refused any intervention.”  “Pain controlled, complaints of pubic bone [pain] but able to sit ambulate and void independently.” |
| Unilateral/authoritarian decisions | “intolerant of vaginal exam”  “SW [social worker] has advised pt [patient] that if there continues to be yelling in room, ACS [adult and child services] will need to be contacted”  patient "placed in bed" |
